# Female rats are more vulnerable to binge drinking behavior in an operant self-administration paradigm: implication for transition to alcohol use disorders

**DOI:** 10.1186/s13293-026-00912-x

**Published:** 2026-04-29

**Authors:** Jérôme Jeanblanc, Amélie Soyer, Mickaël Naassila

**Affiliations:** INSERM UMR1247 - Groupe de Recherche sur l’alcool et les pharmacodépendances GRAP, Université de Picardie Jules Verne, Centre universitaire de recherche en santé CURS, Chemin du Thil, Amiens, 80025 France

**Keywords:** Binge drinking, Alcohol, Sex differences, Operant self-administration, Relapse, Withdrawal

## Abstract

While binge drinking can significantly impact health negatively, it has become increasingly important to understand how sex differences contribute to this hazardous behavior, which may also serve as a risk factor for alcohol use disorder. We employed the binge drinking experimental model we developed previously to specifically analyze sex differences. Forty male and 40 female Long Evans rats were tested in the alcohol self-administration procedure, operationalized as alcohol responding in short daily session. We tested other parameters, including motivation, seeking, responses during cue omission sessions, withdrawal scores, and relapse after abstinence. We also conducted experiments to assess perseverance despite satiety. For the analysis we used first an unsupervised clustering approach using drinking speed and frequency of alcohol responses and then we analyzed our data by taking sex as the differentiating factor. Unbiased clustering analysis revealed four distinct groups: Fast Bingers, Bingers, Extreme Bingers and Low drinkers. Higher alcohol consumption and faster consumption speed correlated with elevated withdrawal scores. Sex-related differences were observed, with females outnumbering males in Extreme bingers. Females also exhibited higher alcohol-seeking behavior, relapse rates, and withdrawal scores. In addition, females exhibit lower sensitivity to devaluation in the satiety test. Our results suggest that females display greater vulnerability to cue-mediated alcohol-seeking behaviors and a more inflexible behavior. This underscores the importance of considering sex as a biological variable in both preclinical and clinical research on binge drinking behaviors that is not only a hazardous behavior but may also be a critical factor in AUD vulnerability, particularly in females.

**Supplementary Information:**

The online version contains supplementary material available at 10.1186/s13293-026-00912-x.

## Abstract (plain language summary)

Binge drinking is a specific pattern of alcohol consumption based on consuming large amounts in a short period of time. Binge drinking is known to be a risk factor to the transition to latter alcohol addiction. A recent study has shown greater brain damage in women who binge drink than in men. Thus, in the present study we used the animal model we developed to mimic the binge drinking behaviour in rats. We used 40 male and 40 female adult rats to evaluate a series of behaviors associated to the consumption of alcohol such as motivation, seeking, relapse and transition to drug addiction. In a first analysis combining both sexes we found 4 distinct groups of drinkers, the bingers, the fast bingers, the extreme bingers and finally the low drinkers that do not drink much alcohol. The distribution of the sexes was not well balanced in the groups with the extreme bingers represented mostly by females. We then evaluated the differences between both sexes and we found that the females consumed more alcohol than the males, females were more sensitive to the contextual cues associated to each delivery of alcohol. We also found that females are more vulnerable to addiction after chronic binge drinking and that they will relapse at higher levels than males. Overall, we demonstrate the need to include the females in our preclinical studies because they do not display similar sensitivity to drug consumption than males.

**Supplementary Information:**

The online version contains supplementary material available at 10.1186/s13293-026-00912-x.

## Highlights

Cluster analysis based on quantity of alcohol consumed during a 15-min operant self-administration session and speed of consumption revealed 3 different groups of ‘bingers’ and a group of ‘low drinkers’

The ‘extreme bingers’ differ from the others by the quantity consumed and the ‘fast bingers’ differ from the ‘bingers’ by consuming the same amount of alcohol but twice faster

The ‘extreme bingers’ group is composed by more than 70% of female rats

The sex-based analysis demonstrates a higher sensitivity to the cue associated to the delivery of alcohol in females

Females are less sensitive to the devaluation of the reward than the males leading to perseverance of the operant behavior despite satiety and higher levels of relapse after abstinence

**Supplementary Information:**

The online version contains supplementary material available at 10.1186/s13293-026-00912-x.

## Introduction

Binge drinking is a dangerous and harmful alcohol consumption behavior characterized, among other criteria, by large quantities ingested over a short period of time [1]. Unlike alcohol use disorder (AUD), which is defined as a clinical condition characterized by impaired control over alcohol use and other diagnostic criteria, binge drinking refers to a specific pattern of alcohol consumption characterized by episodic heavy drinking leading to acute intoxication. Importantly, while some individuals with AUD may engage in binge drinking episodes, binge drinking itself is not considered a diagnostic category of AUD [2]. Binge drinking behavior is rather heterogeneous behavior. We recently proposed a mathematical model to better categorize binge drinking behavior in young adults, and we identified the existence of four groups based on the consumption profile: low-risk, hazardous, binge, and high-intensity binge drinking [3]. Furthermore, binge drinking is considered a factor involved in the development of AUD. A study in France has shown that frequent binge drinking between 18 and 25 years (4+/5 + drinks/occasion for females/males, more than twice a month) leads to an approximately threefold increased risk of developing AUD at adulthood (between 25 and 45 years) [4]. Another study in the United States showed that nearly 80% of those young adults who reported engaging in high-intensity drinking (10 + drinks in a row) at age 29/30 later reported AUD symptoms at age 35 [5]. Therefore, it is conceivable that a chronic pattern of binge drinking is associated with the transition to addictive behavior, and it is possible, in particular, that the repetitive aspect of binge drinking behavior facilitates the establishment of habit learning or attentional biases, that may play an important role in AUD.

While AUD is more commonly found in men, there is a notable and accelerating rise in the number of women affected by AUD, particularly among adolescents [6]. Women exhibit greater susceptibility to the medical consequences associated with alcohol consumption, such as alcohol-related liver disease. This trend is not solely attributed to sex-based differences in alcohol pharmacokinetics; women also demonstrate an expedited progression from initial alcohol use to the onset of AUD. The current knowledge on binge drinking behavior has not clearly demonstrated sex-related differences in vulnerability, not only to this behavior but also regarding its role in the development of AUD or some of its symptoms. However, some studies have shown sex-related differences in young binge drinkers. For example, one study that used clustering analysis, suggested that male and female binge drinkers should not be considered a unitary group, but rather a population of individuals that encompasses at least 2 distinct personality patterns with Cloninger’s type I (high harm-avoidance) and II (high novelty-seeking) AUD typology [7].

Various animal models have been developed to study binge drinking behavior [8]. We demonstrated that daily access to an alcohol self-administration operant chamber for just 15 min over several months facilitates the emergence of a behavior resembling binge drinking in humans, with rapid consumption leading to significant blood ethanol levels and ataxia in rats [9]. In addition, we identified using the same model, that binge drinking behavior is dependent upon genetic factors since among three different outbred rat strains, the Long Evans one is more prone to binge drinking [10]. This model is also characterized by excellent predictive validity, as we have demonstrated that various treatments for AUD are effective [11].

In both human and animal studies, binge drinking is typically operationalized using behavioral measures combining the quantity of alcohol consumed and the temporal pattern of intake rather than direct measurement of blood alcohol levels. Therefore, reducing the time required to access alcohol, as observed during happy hours in humans, acts as a facilitator of binge drinking behavior.

It is also important to emphasize that any direct comparison between paradigms requires isomorphic contingencies—namely equivalent session duration, response cost, delivery granularity, and mode of access (continuous sipper vs. unitary drops). In the absence of such equivalence, reducing session length in a sipper model primarily decreases the number of opportunities to respond and the total available volume, whereas in an FR-3 schedule, the same temporal compression intensifies early responding and promotes front-loading. This distinction is critical, as it is precisely this front-loading dynamic that operant binge models are designed to capture.

Here, we sought to characterize sex differences in chronic voluntary binge drinking and especially in several operant self-administration criteria such as ingested alcohol, drinking speed, alcohol seeking in a drug omission session and motivation. Reward comprises learning (cue associations), hedonic (“liking”), and motivational (“wanting”) components [12]. Initially linked with a reward, conditioned stimuli have the potential to transform into independent motivational cues, prompting both appetitive approach and consummatory behaviors [12]. The transition from casual to compulsive alcohol use is believed to coincide with heightened motivation to get al.cohol. In animals, this escalated exertion of effort can be quantified through progressive ratio (PR) schedules of reinforcement. We also measured other criteria more linked to addiction such as withdrawal symptoms after abstinence and perseverance of the operant self-administration despite the devaluation of the reward by a satiety test. Thus, and as we recently did in humans, we used first a clustering approach to better characterize different binge drinking patterns and since the results revealed sex-related differences we further determined criteria related to addictive behaviors in both sexes.

## Materials and methods

### Animals

**Fig. 1:**
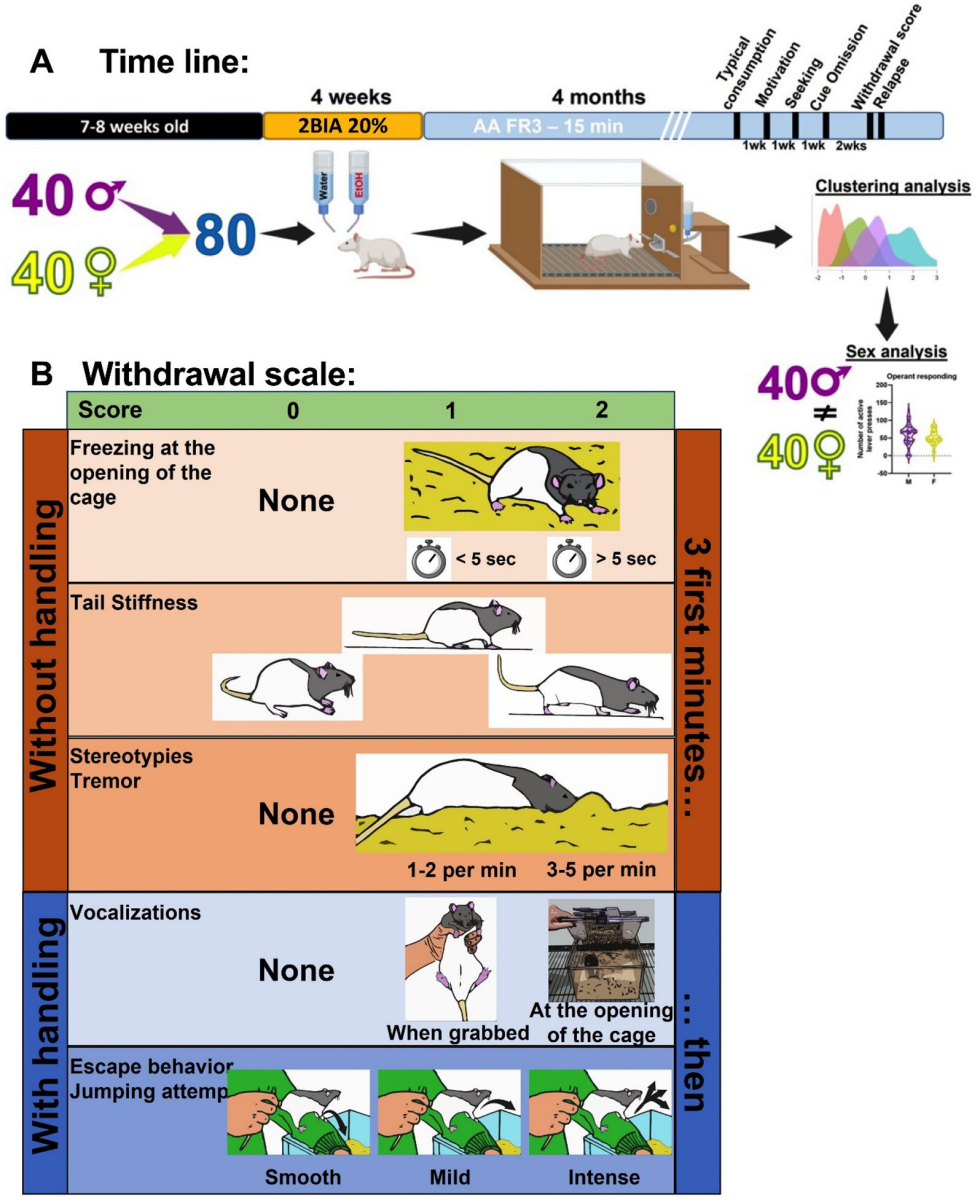
**A**. Timeline of the experiment and analysis. The 80 rats underwent our operant binge drinking (OBD) protocol and were subsequently tested for various parameters relevant to the study of Alcohol Use Disorder (AUD). A clustering analysis was performed focusing on the quantity of alcohol consumed in one session of OBD and the speed of consumption (time to reach 50% of the total number of active lever presses). All the different parameters studied were analysed based on the four clusters identified. Subsequently, a sex-based analysis was conducted on the same parameters. **B**. Withdrawal score scale. To assess the withdrawal score, we followed a scale divided into 5 items with scores. The first three items are based on simple observation without manipulation. Open the cage and observe the rat for 3 min. At the opening, if the rat moves normally, count 0; if it freezes, count 1 if it is less than 5 s and 2 if more than 5 s. Observe the tail and its orientation (1 if horizontal during the walk, 2 if vertical to the top). Then, observe if the rat exhibits tremors or stereotypies. The most frequent stereotypies are first burying its head in the bedding and then grooming. Depending on the frequency of the stereotypies, score 0 for none, 1 if 1–2 per minute, and 2 if 3 or more per minute. If the rat vocalizes at the opening of the cage, score 2; if it only vocalizes during manipulation, score 1; if it does not vocalize at any time, score 0. Finally, we evaluated escape behaviour by placing the rat on our arm and assessing the way the rat returns to its cage. If the descent is smooth, score 0; if the rat jumps, score 1; if the rat jumps repetitively as soon as its paws touch your arm, score 2

Forty males and forty females Long Evans rats were purchased from Janvier Labs (Le Genest-Saint-Isle, France) at the age of 7 weeks. The time line of all experiments is presented on Fig. 1A. The animals were housed individually without enrichment in a thermo-regulated animal facility (22 ± 1 °C and 40 ± 10% humidity), with a 12/12hrs light/dark cycle (light on at 8:00 am). All animals were provided with water and food *ad libitum*. The behavioral tests were performed between 9:00 and 12:00 pm. The experimental procedures were performed according to the principles set forth by the Council responsible for the protection of animals used for experimental purposes (EEC N° 86/609) and approved by the local ethical committee (CREMEAP, APAFIS#26615).

### Reagents

Ethanol, (ETOH 96°) was purchased from VWR (Strasbourg, France) and was diluted in tap water at the concentration of 20% (v/v).

### Voluntary operant binge drinking

The procedure of voluntary operant binge drinking is identical to the one already described (9). Briefly, rats were first submitted to 4 weeks of a two-bottle choice intermittent access paradigm with water solution available every day and with 20% EtOH solution available every other day (Mondays, Wednesdays and Fridays) for 24 h starting at 2:00 pm. At the end of this procedure, rats underwent training sessions of operant self-administration for EtOH (20% v/v) in Med Associates operant cages (ENV-008, Med Associates, Albans, VT, USA). Two overnight sessions under a fixed ratio 1 (FR1) schedule were followed by five consecutive sessions under each of the following conditions: 5 × 1 h–FR1, 5 × 1 h–FR3, 5 × 30 min–FR3. Duration was finally reduced to 15 min until the end of the experiment. Maintenance of binge drinking behavior was achieved over 2 months (5 sessions a week). Ethanol reward was a 0.1 mL drop of the 20% ethanol solution and its delivery was accompanied by contextual cues: a light (within the delivery compartment) and a tone stimulus both for 3 s. The cages are controlled and the behaviors recorded through the MEP IV software (Med Associates, Albans, VT, USA).

### AUD-associated behaviors

#### Typical session of ethanol consumption

After about 8 weeks of training and stabilization rats were kept on the same schedule of 15-min sessions 5 days a week. Once the behavior is stable for 2 weeks in a row, we started to collect the data and perform all the different tests. For the typical session of ethanol, we choose to use a Wednesday to avoid the rebound of consumption we usually observe on Mondays and the decrease observed on Tuesdays. During this first Wednesday of the behavioral tests, we collected the number of active lever presses, the number of inactive lever presses and the time for each press and each delivery allowing us to analyze the quantity and the speed of consumption. Rats were weighted the day of the test to calculate the exact amount of pure ethanol consumed expressed in g/kg of bodyweight. Drinking speed was defined as the time required for each animal to reach 50% of its total ethanol intake during the session. This parameter was chosen to capture the temporal dynamics of alcohol consumption independently of total intake. In binge-drinking models, rapid early intake (“front-loading”) is considered a key behavioral feature of excessive alcohol consumption. The time-to-50% metric therefore provides an index of front-loaded drinking behavior rather than simply reflecting response rate.

#### Motivation

Increased motivation to obtain a drug, often reflected by a greater willingness to work for it, is commonly considered a hallmark of addictive behavior across substances. It is thought to reflect, at least in part, the transition from controlled or recreational intake to more compulsive patterns of drug seeking, and therefore represents an important dimension of addiction-like phenotypes [13]. After ten sessions under a fixed-ratio 3 (FR3) 15-min reinforcement schedule, animals’ motivation to self-administer alcohol or saccharin was assessed using a progressive-ratio reinforcement protocol. In this procedure, the response requirement for each subsequent reward gradually increases (3, 4, 5, 7, 9, 12, 15, 17, 20, 22, 25, 28, 30, 33, and 35 responses) [14]. Eventually, animals cease pressing the active lever; this final ratio completed is termed the breaking point, and serves as an index of the animal’s motivational drive to obtain ethanol. Sessions lasted 30 min to ensure sufficient time for animals to complete the progressive schedule [14].

#### Seeking (drug omission test)

Drug seeking in the absence of drug availability is widely used as an operational measure related to craving and reduced behavioral control in animal models. In our protocol, alcohol seeking was evaluated during a dedicated session in which no alcohol solution was delivered despite active lever presses, while the conditioned cues previously associated with alcohol remained present. Persistence of active lever pressing under these omission conditions is generally interpreted as reflecting increased motivation to obtain alcohol and is often considered indicative of craving-like behavior or reduced control over alcohol seeking [15].

#### Cue omission test

One of the behavioral criteria of addiction is the excessive sensitivity to environmental cues associated with drug consumption, which can by themselves trigger or intensify drug-seeking behavior. The Cue Omission test is useful for assessing this cue reactivity, namely the disproportionate motivational value acquired by stimuli predicting reward delivery [16, 17]. Although this test does not directly measure attentional capacities or attentional biases in the strict cognitive sense, it provides insight into the salience of drug-associated cues and the degree of behavioral automatization: a strong cue-controlled response is typically observed in addictive-like behaviors. In our operant model of binge drinking, the cues associated with solution delivery gradually acquire secondary reinforcing value. It is well established that such contextual cues can elicit consumption-related behaviors. Therefore, to evaluate the vulnerability to these cues in our model, we conducted a Cue Omission session. Sensitivity to the cue associated with delivery was assessed in a session in which the drug was still delivered after three active lever presses, but without presentation of the cues normally paired with its delivery. In our paradigm the drug is still delivered after 3 active lever presses but no cues were associated with it.

#### Relapse after chronic binge drinking

After 11 weeks of operant binge-drinking sessions, rats underwent an ethanol deprivation period during which they were not exposed to the operant self-administration chambers for 10 consecutive days. Following this abstinence interval, animals were reintroduced into the operant chambers for a standard binge-drinking session in which alcohol-associated cues were presented and ethanol was again available. To facilitate the initiation of relapse, a priming free drop of ethanol was delivered at the onset of the session. This procedure enables the assessment of relapse-like drinking behavior following chronic intermittent alcohol exposure.

#### Withdrawal score

Withdrawal syndrome refers to the emergence of physical signs following drug cessation and is a hallmark of dependence (signs of physical dependence in AUD). The withdrawal scale used in this study (Fig. 1B) was adapted from the scale originally described by Gilpin et al. ^18^, which was developed to assess withdrawal severity in alcohol-dependent animals exposed to alcohol vapor. Our adaptation was designed to allow assessment of withdrawal-like signs across different patterns of alcohol exposure, including chronic drinking, binge-like drinking, and dependence. The procedure was also slightly modified to prioritize an initial observation period without handling and to simplify the scoring by selecting a limited number of clearly identifiable behavioral items. In addition, some items of the original scale were adjusted or removed when they showed high variability or limited relevance under our experimental conditions. After five days of abstinence, rats were observed for several minutes in their home cage, and their behavior was evaluated according to the following criteria: (i) aggression and/or vocalizations; (ii) posture (immobility or slowing); (iii) tail rigidity; (iv) hyperlocomotion (e.g., convulsions or escape attempts); and (v) tremors, stereotypies, or rotational behavior. Each criterion was rated on a 0–2 scale based on symptom intensity, and individual scores were summed to generate a global withdrawal score.

As a control for this scale evaluation, we generated a new cohort of 10 females and 10 males trained to self-administer sucrose (2%) following the exact same protocol that the one used for the ethanol operant self-administration (2BCIA for 4 weeks, overnight sessions, same schedules and durations shifts).

#### Resistance to devaluation (satiety test)

Resistance to outcome devaluation, i.e. the persistence of consummatory behavior despite a reduced reward value, is a key marker of the transition from controlled to compulsive drug use. In addiction models, animals that remain goal-directed typically decrease their responding when the drug is devalued, whereas animals that have shifted toward habitual or addiction-like behavior show little or no reduction. To assess whether our animals displayed such a transition, we implemented a satiety-induced devaluation procedure. After several days of stable operant self-administration, rats received 1 h of free access to ethanol in their home cage (two-bottle choice: water vs. ethanol) to reduce the reward value of the drug. Immediately after this devaluation session, animals were placed back into the operant chambers for a standard 15-min FR3 session with alcohol and associated cues available. The number of active lever presses during this session served as the index of sensitivity, or resistance, to reward devaluation.

### Statistical analysis

#### Clustering

BD phenotype analysis is performed to obtain different clusters. In a first step of analysis, and as it is done in humans [19], we combined the 80 rats in this analysis without separating males and females. Then in a second set of analysis, we performed an identical k-means analysis in both males and female separately. The analysis is performed using the JASP software (Version 0.14.1) using k-means partitioning. This method belongs to the unsupervised classification algorithms which means that the groups do not exist before being created so the number of clusters is determined during the analysis. The optimal number of clusters was determined using complementary criteria including the elbow method (within-cluster sum of squares), Bayesian Information Criterion (BIC), Akaike Information Criterion (AIC), and silhouette coefficient. Converging results across these metrics indicated that a four-cluster solution provided the best balance between model fit and interpretability. Two parameters are chosen for the classification method from the “typical session of self-administration: the ethanol consumption (in g/kg) and the speed of consumption calculated as the time to obtain 50% of the total rewards. From the different groups obtained thanks to these criteria, the severity of the addictive behavior (motivation, seeking, relapse) is analyzed for each group.

#### Post-clustering and sex effect statistical analyzes

All data are presented as means ± standard error (SEM). Data were analyzed (Sigma Plot) using the Shapiro-Wilk test for normality. When normality and homogeneity of the variances were met a one-way or two-way ANOVA test (with or without repeated measures) followed by a Tukey multiple comparison test when a significant effect was observed. For single comparison, we used a Student’s *t* test (two-tailed), and for correlation, the Pearson correlation test is used. When the data do not follow a normal distribution, there were analyzed using a Kruskal-Wallis test followed by a Dunn’s test for multiple comparison or a Mann-Whitney test for simple comparisons. Significance is established at *p* < 0.05.

## Results

### Clustering analysis

First, a clustering analysis is performed using the k-means method on the data from the 80 rats exposed to EtOH. It allows us to group similar data in the same cluster. Then a screen plot is performed which compiles the ratio between the total within cluster sum of squares and the total sum of square. This allows us to locate the “elbow”, which corresponds to the ideal number of clusters (in our case, 4 groups), see Additional Fig. 1 and Additional Table 1. The selected model is the one that minimizes the Bayesian information criterion (BIC), derived from the Akaike information criterion (AIC). The best model has the lowest BIC and AIC values. Our selected model has the highest silhouette coefficient, corresponding to the clustering quality. Moreover, the elevated r^2^ (0.752) suggests a good fit of the model.

**Fig. 2:**
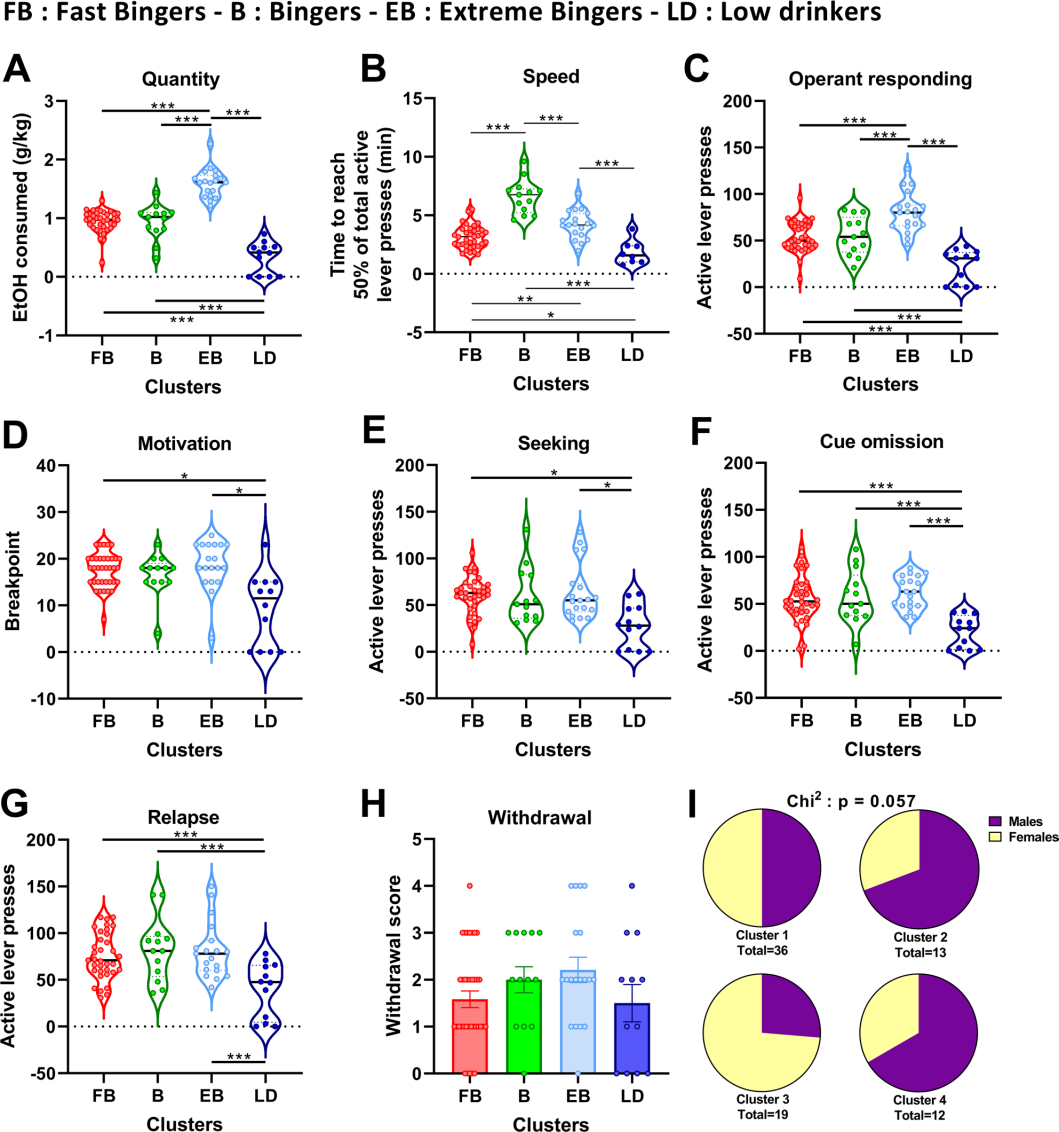
Clustering analysis. Based on the quantity of alcohol consumed (**A**) and the speed of consuming it (**B**), the clustering analysis provided 4 distinct groups of consumers: FB = Fast Bingers, B = Bingers, EB = Extreme bingers and LD = Low Drinkers. The operant responding (**C**) during a typical session of OBD was analyzed depending of these clusters, as well as the motivation (**D**) through a progressive ratio session, the seeking (**E**) for alcohol (a session in which alcohol is absent but the cues associated are present), the perseverance of operant responding during a session in which alcohol is delivered but the cue-associated are absent (**F**, cue omission) and finally the relapse (G) after 14 days of abstinence. The bold black dotted line within each violon box indicates the median and the fine dot-colored lines represent the quartiles. The withdrawal score (**H**) was measured the day before the relapse session meaning 13 days after the last session of OBD. Finally, the distribution of both sexes within each cluster is depicted in panel (**I**). FB *n* = 36, B, *n* = 13, EB *n* = 19, LD *n* = 12. One-way ANOVA followed by Tukey test: * *p* < 0.05, ** *p* < 0.01, *** *p* < 0.001

Four groups are obtained from this model (Fig. 2; Table 1):

- Group 1, consisting of 18 ♂ and 18 ♀, considered ‘fast Bingers’ with EtOH consumption at 0.94 g/kg and time to 50% reward at 3.14 min.
- Group 2, consisting of 9 ♂ and 4 ♀, considered ‘Bingers’ with EtOH consumption at 0.95 g/kg and time to obtain 50% of rewards at 6.59 min.
- Group 3, consisting of 5 ♂ and 14 ♀, considered ‘extreme Bingers’ with EtOH consumption at 1.60 g/kg and the time to obtain 50% of the rewards at 4.20 min.
- Group 4, consisting of 8 ♂ and 4 ♀, considered the ‘low drinkers’ with EtOH consumption at 0.33 g/kg and time to obtain 50% of the rewards at 1.21min.

#### AUD-associated behaviors

The statistical analysis of the different AUD-associated behaviors is listed in Table 2.

The consumption averaged over the 3 last sessions of drinking in the 2BCIA procedure indicates that the 3 groups categorized as ‘bingers’ (Group 1, 2 and 3) do not differ between them but are all significantly higher drinker than the group 4 (Table 1).

**Table 1:** AUD-associated behaviors in the 4 identified groups of the whole population (males and females)

AUD-associated behaviors	ANOVA or Kruskal Wallis	G1 Fast bingers *n* = 18 F + 18 M	G2 Bingers *n* = 4 F + 9 M	G3 Extreme bingers *n* = 14 F + 5 M	G4 Low drinkers *n* = 4 F + 8 M	Post hoc pairwise comparison
2BC consumption(g/kg/24hr)	F_(3,76)_ = 6.23 *p* < 0.001	7.39 ± 0.5 **	6.09 ± 0.82	8.33 ± 0.62 ***	4.73 ± 0.73	G3 = G1=G2(G3 = G1)> G4
SA – Active lever presses	F_(3,76)_ = 24.496 *p* < 0.001	53.69 ± 2.72***	55.00 ± 5.67***	80.32 ± 5.00***	22.33 ± 5.14	G3 > G2 = G1 > G4
SA – EtOH consumed (g/kg/15 min)	H = 56.17 *p* < 0.001	0.94 ± 0.03 ***	0.94 ± 0.08 ***	1.60 ± 0.06 ***	0.33 ± 0.08	(G3 > G2=G1)> G4
Speed of consumption (time to achieve 50% of total consumption - min)	F_(3,76)_ = 50.68 *p* < 0.001	3.14 ± 0.16 ***	6.59 ± 0.40 ***	4.20 ± 0.28 ***	1.21 ± 0.35	G4 > G1> G3 > G2
Motivation (active lever presses)	H = 16.53 *p* < 0,001	85.51 ± 4.78 ***	81.38 ± 8.43	96.84 ± 8,97 ***	40.67 ± 11.38	G3 > G1 = G2 > G4
Seeking (active lever presses)	H = 15.60 *p* < 0,001	60.69 ± 3.33 ***	59.62 ± 8.31	63.84 ± 7.04 ***	27.42 ± 6.70	(G3 = G1=G2)> G4
Cue Omission (active lever presses)	F_(3,76)_ = 9,619 *p* < 0,001	55.61 ± 4.04 ***	57.38 ± 7.85 ***	61.89 ± 4.01 ***	20.50 ± 4.87	(G3 = G2=G1)> G4
Relapse (g/kg/15 min)	F_(3,76)_ = 9.99 *p* < 0.001	1.38 ± 0.07 ***	1.34 ± 0.15 **	1.62 ± 0.11 ***	0.67 ± 0.15	(G3 = G2=G1)> G4
Withdrawal Score (arbitrary unit)	F_(3,75)_ = 1.61 *p* = 0.195	1.60 ± 0.18	2.00 ± 0.28	2.21 ± 0.27	1.50 ± 0.40	G1 = G2=G3 = G4

Normality was evaluated if validated, an ANOVA (F value in the second column) followed by a Tukey test for multiple comparison was perform on the data. If Normality was not validated, a Kruskal-Wallis test was performed (H value in the second column) followed by a Dunn’s test for multiple comparison. * p < 0.05; ** p < 0.01; *** p < 0.001 vs. ”Low drinkers”. In the “Post hoc pairwise comparison” column, groups are placed from the higher to the lowest even if not different statistically. The symbol “>” indicate a significant difference while a symbol “=” indicates no significant difference between the groups

After prolonged training to self-administer alcohol, the group ‘extreme bingers’ significantly drink more pure ethanol than all other groups (Fig. 2A). ‘Fast bingers’ (Group 1) consume the 50% of their total amount faster than the ‘bingers’ (Group 2) but not than the ‘extreme bingers’ (Group 3) and ‘low drinkers’ (Group 4) (Fig. 2B). Profiles of operant responding, meaning the number of active lever presses, are similar to the quantity of ethanol consumed (Fig. 2C). Motivation (Fig. 2D), seeking (Fig. 2E), response during a cue omission test (Fig. 2F) and relapse after abstinence (Fig. 2G) are similar between the 3 bingers groups and all 3 groups are significantly higher than the “low drinkers”. When cue-omission responding was compared to baseline operant responding, only modest reductions were observed, with the most noticeable decrease in the Extreme Bingers group. The 2-way ANOVA with repeated measures revealed a significant effect of the clusters (F_(3, 76)_ = 19.51, *p* < 0.001 but not of the sessions (F_(1, 76)_ = 2.42) and an interaction between both factors (F_(3, 76)_ = 4.39, *p* < 0.01). The post-hoc analysis revealed a significant difference only within the cluster Extreme bingers (*p* < 0.001). During the period of abstinence, withdrawal score was measured and we found no significant differences between the 4 groups (Fig. 2H). Within each of the clusters, the sex distribution is relatively different (Chi-square = 7,520 with 3 degrees of freedom. (*p* = 0,057)) with 50% of males in the ‘fast bingers’ group, more than 60% of males in the ‘bingers’ group and more than 70% of females in the ‘extreme bingers’ group. We finally found a majority of males in the ‘low drinkers’ (Fig. 2I).

To ensure the robustness of our model, we run a new k-means analysis within each sex. It is noteworthy that the unsupervised analysis provided the exact same 4 clusters (Additional Fig. 2). We then performed an identical analysis for each of the behaviors for the clusters found in males (Additional Fig. 3 and Additional Table 1) and Females (Additional Fig. 4 and Additional Table 2).

**Fig. 3:**
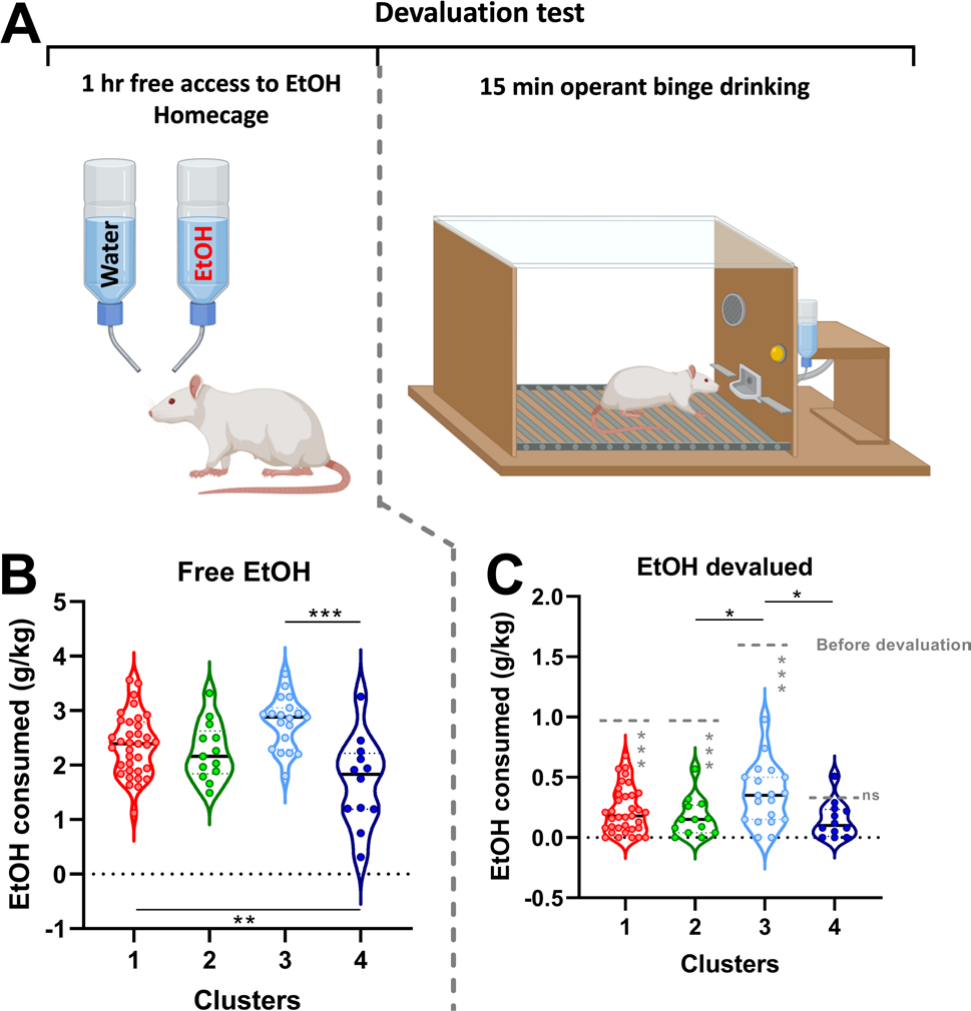
Perseverance despite devaluation. (**A**) The devaluation test is divided in 2 phases: the first one consists in providing free alcohol for one hour in the home cage of the rats. The second phase is the phase of test in which rats are submitted to a regular OBD session with access to alcohol upon 3 consecutive active lever presses. In Panel (**B**) are depicted the amount of alcohol consumed (expressed in g/kg/1 hr) during the hour of free access to alcohol. The result of the devaluation is depicted in panel (**C**) with the amount of alcohol consumed (g/kg/15 min) during the OBD session before devaluation (grey dotted lines) and after devaluation for each cluster. Cluster 1 *n* = 36, Cluster 2, *n* = 13, Cluster 3 *n* = 19, Cluster 4 *n* = 12. One-way ANOVA followed by Tukey test: * *p* < 0.05, *** *p* < 0.001. Within each cluster, before vs. after devaluation comparisons were performed using paired Student’s t-tests and are indicated by grey asterisks (*p* < 0.001) or not significant (ns)

In order to evaluate if rats did their transition from controlled to uncontrolled behavior, we performed, as already mentioned, a satiety test (Fig. 3A). During the free hour of access to alcohol in the homecage, we only found one statistical difference between the ‘extreme bingers’ and the ‘low drinkers’ that drink less (Fig. 3B). After devaluation, during a regular FR3-15 min session of self-administration we observed a higher consumption in the ‘extreme bingers’ as compared to the ‘bingers’ and ‘low drinkers’ (Fig. 3C).

#### Sex differences

**Fig. 4:**
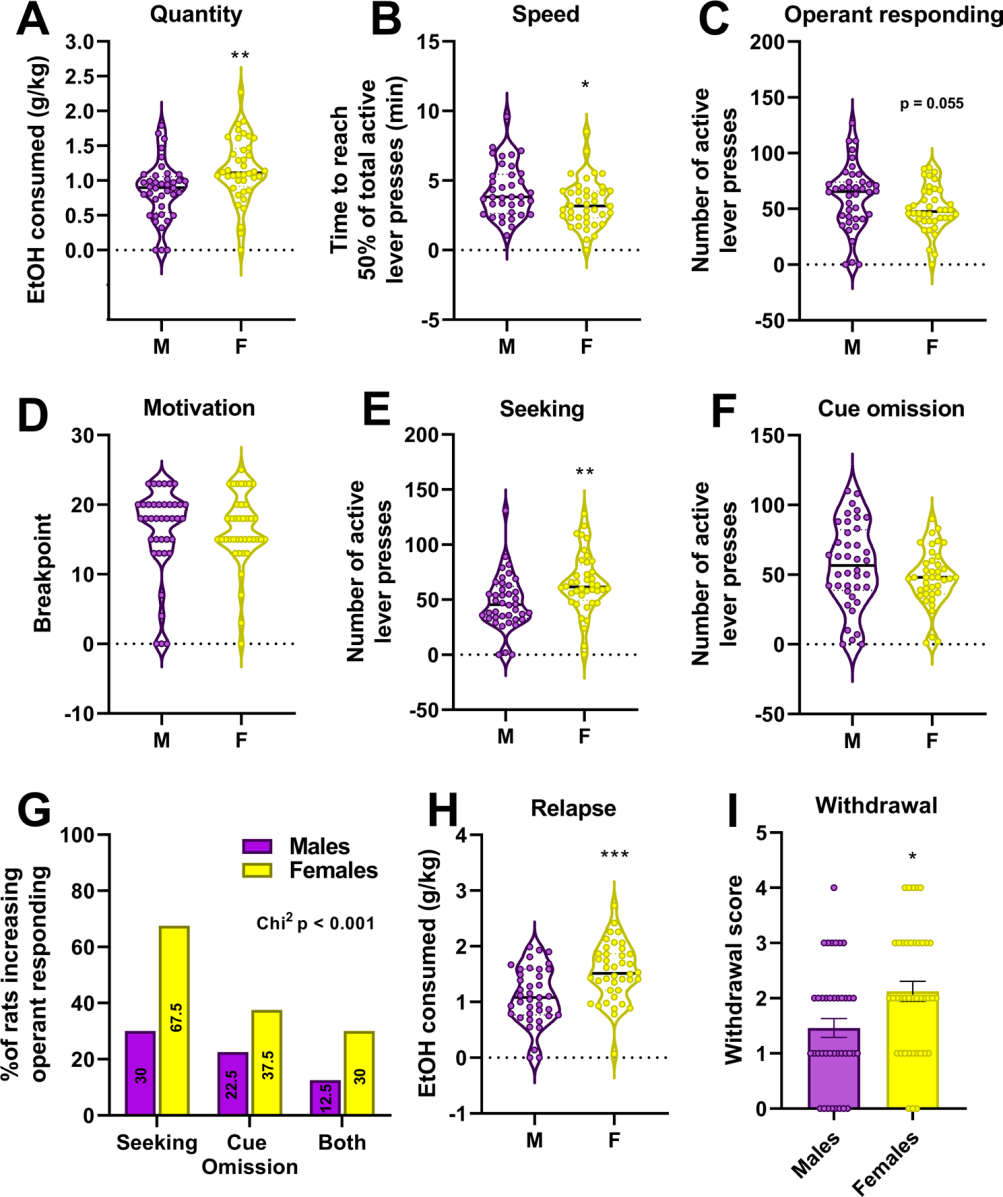
Sex-based analysis. Based on the sex, quantity of alcohol consumed (**A**), speed of consuming it (**B**), operant responding (**C**) during a typical session of OBD the motivation (**D**) through a progressive ratio session, the seeking (**E**) for alcohol (a session in which alcohol is absent but the cues associated are present), the perseverance of operant responding during a session in which alcohol is delivered but the cue-associated are absent (**F**, cue omission). We analysed the proportion of individuals that increase their operant responding during the Seeking and the Cue Omission sessions as compared to the typical OBD session (**G**). Relapse (**H**) after 14 days of abstinence was also analysed. The withdrawal score (**I**) was measured the day before the relapse session meaning 13 days after the last session of OBD. Finally, the distribution of both sexes within each cluster is depicted in panel **I**. Bilateral unpaired Student’s t-test for **A**,** B**,** C**,** D**,** E**,** F**,** H** and I: * *p* < 0.05, ** *p* < 0.01, *** *p* < 0.001. Chi-square for G. The bold black dotted line within each violon box indicates the median and the fine dot-colored lines represent the quartiles

We identified that there was some discrepancy in the sex balance in some of the groups an especially in the group “Extreme Bingers”, thus we analyzed the data based on the sex. The results are depicted in Fig. 4 and the statistical analysis of these data in Table 2.

**Table 2:** AUD-associated behaviors in males and females. * *p* < 0.05; ** *p* < 0.01; *** *p* < 0.001 between Males and Females. The last column indicates which group shows higher values for each of the behaviors

AUD-associated behaviors	Student’s t-test	Males	Females	% of change vs. Males	
2BC consumption (g/kg/24hr)	*p* = 0.046	6.41 ± 0.38	7.60 ± 0.45 *	18%	F > M
SA – Active lever presses	*p* = 0.055	60.95 ± 4.58	50.10 ± 3.19		M > F (ns)
SA – EtOH consumed (g/kg/15 min)	*p* = 0.003	0.86 ± 0.07	1.16 ± 0.07 **	35%	F > M
Speed of consumption (time to achieve 50% of total consumption - min)	*p* = 0.043	4.28 ± 0.31	3.45 ± 0.27 *	-20%	F > M
Motivation (active lever presses)	*p* = 0.744	82.30 ± 6.03	79.58 ± 5.73		M = F (ns)
Seeking – Drug Omission (active lever presses)	*p* = 0.004	47.60 ± 3.90	64.95 ± 4.30 **	36%	F > M
Cue Omission (active lever presses)	*p* = 0.139	56.43 ± 4.78	47.83 ± 3.20		M > F (ns)
Relapse (g/kg/15 min)	*p* = 0.0003	1.10 ± 0.08	1.54 ± 0.08	40%	F > M
Withdrawal Score (arbitrary unit)	*p* = 0.009	1.46 ± 0.17	2.13 ± 0.18 **	46%	F > M
Satiety 1 h free EtOH consumed (g/kg/1 hr)	*p* = 0.0017	2.11 ± 0.10	2.57 ± 0.10 **	22%	F > M
Satiety EtOH consumed post devaluation (g/kg/15 min)	*p* < 0.001	0.12 ± 0.02	0.34 ± 0.04 ***	183%	F > M

First during the 2BCIA procedure, the average of alcohol consumed over the 3 last drinking sessions, females consumed significantly more ethanol than males (7.60 ± 0.45 vs. 6.41 ± 0.38 g/kg/24 hrs, *p* < 0.05, Table 2). After weeks of stabilization of the operant behaviour, female rats consumed more ethanol than males (Fig. 4A) and do it faster (Fig. 4B) without exhibiting higher number of active lever presses (Fig. 4C). Despite this higher consumption, motivation measured as the breaking point during a progressive Ration test is not different between males and females (Fig. 4D). We thus evaluated the sensitivity to the presence or absence of the drug and of the associated cues. During the seeking session, in which no alcohol is provided despite the active lever presses and the presence of the cues, the females significantly pressed more than the males (Fig. 4E). On the opposite, when drug was delivered but no cues were associated to the delivery, no difference between both sexes (Fig. 4F). During these 2 sessions, some rats showed an increase in active lever presses as compared to baseline responding (3 consecutive self-administration sessions). Interestingly, for the “seeking” as well as for the “cue omission” sessions higher proportion of females exhibited this increase (67.5% vs. 30% for the “seeking sessions” and 37.5% vs. 22.5% for the “cue omission” session). 30% of female rats increased their pressing levels in both sessions (seeking and cue omission) whereas only 12.5% of males did (Fig. 4G, Chi^2^*p* < 0.001). After a prolonged abstinence, females rats showed higher level of relapse than males ((Fig. 4H). During this abstinence period, female rats exhibited also higher withdrawal score than males (Fig. 4I). Specifically concerning this score of withdrawal, females displayed higher withdrawal scores than males (female mean ± SD 2.18 ± 1.13; male 1.43 ± 1.08; Mann–Whitney *p* = 0.0043; Cohen’s d = 0.68). Item-wise analyses indicated that this sex effect was primarily driven by higher tail stiffness (female 0.78 ± 0.42 vs. male 0.48 ± 0.55; Holm-adjusted *p* = 0.023) and escape/jumping attempts (female 0.35 ± 0.48 vs. male 0.05 ± 0.22; Holm-adjusted *p* = 0.0044), whereas vocalizations and tremor/stereotypies did not differ robustly. Importantly, sucrose control rats had near-zero scores with no sex difference (*p* = 0.30), and ethanol exposure markedly increased scores versus controls within each sex (females *p* = 3.0 × 10⁻⁵; males *p* = 2.8 × 10⁻⁴). See Additional Table 3 and Additional Figs. 5, 6 and 7. In addition, withdrawal scores showed a weak association with drinking speed when both sexes were analyzed together (R² = 0.0495, *p* = 0.055). When analyzed separately, a modest but significant correlation was observed in females (R² = 0.1014, *p* = 0.048), whereas no significant relationship was detected in males.

**Fig. 5:**
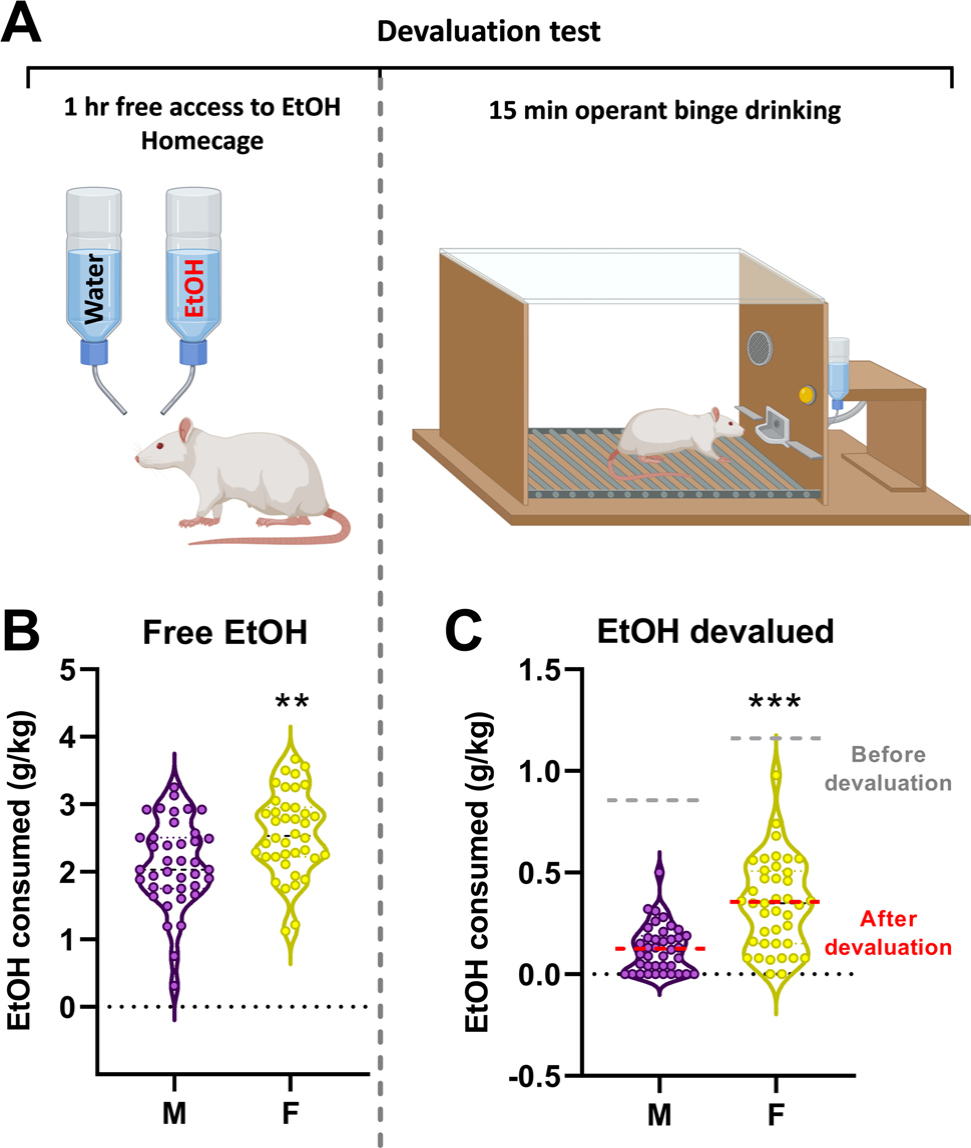
Females persevere more despite devaluation. (**A**) The devaluation test is divided in 2 phases: the first one consists in providing free alcohol for one hour in the homecage of the rats. The second phase is the phase of test in which rats are submitted to a regular OBD session with access to alcohol upon 3 consecutive active lever presses. In Panel (**B**) are depicted the amount of alcohol consumed (expressed in g/kg/1 hr) during the hour of free access to alcohol. The result of the devaluation is depicted in panel (**C**) with the amount of alcohol consumed (g/kg/15 min) during the OBD session. Bilateral unpaired Student’s t-test for A, B, ** *p* < 0.01, *** *p* < 0.001

In regard to the perseverance of instrumental responding despite reward devaluation, we performed, as previously described, a satiety test to assess whether animals would reduce their lever pressing when the value of the ethanol reward was diminished (Fig. 5A). During the free access period to alcohol in the homecage, female rats consumed significantly more alcohol than males (+ 22%, Fig. 5B). Interestingly, this higher intake persisted in the subsequent operant self-administration session, during which female rats consumed drastically more alcohol than males (+ 183%, Fig. 5C).

## Discussion

Binge drinking is a heterogeneous pattern of alcohol consumption characterized by multiple dimensions, including the speed, quantity, and frequency of intake, as well as associated consequences such as blackout episodes and the severity of hangovers. Building on our previous results on binge drinking behavior in students showing the existence of four subgroups [3], we applied a similar type of clustering analysis to our animal model of operant binge drinking, which we have developed and begun to characterize [11, 20] ,although without initially considering sex differences.

Our cohort of 80 rats (40 females and 40 males) was trained to self-administer an ethanol solution during short, binge-like access periods, providing a robust and sex-balanced model of excessive alcohol intake. After a minimum of eight weeks of training, this longitudinal design allowed us to establish stable baseline consumption levels and to systematically evaluate a comprehensive set of parameters associated with AUD. Our first unsupervised clustering analysis based on criteria of alcohol quantity and consumption speed identified, similar to our clinical study [3], four distinct groups. Importantly, the animals classified as “bingers” consumed ethanol levels above the threshold previously associated with pharmacologically relevant intoxication in this operant model. Although the clustering analysis relied on only two behavioral dimensions (ethanol intake and speed of consumption), these variables were specifically chosen because they capture two fundamental aspects of binge drinking: quantity and temporal dynamics of intake. In addition to the quantity consumed, the temporal dynamics of intake are critical for defining binge drinking. The time required to reach 50% of total intake provides an index of front-loading behavior, which has been described as a core feature of binge-like drinking patterns in rodents.

Importantly, the resulting clusters showed clear behavioral differences across multiple independent measures, including motivation, seeking, relapse, and resistance to devaluation. This convergence across behavioral dimensions supports the biological relevance of the clustering solution and suggests that these phenotypes reflect meaningful variability in binge-drinking trajectories rather than statistical artifacts.

It is noteworthy that during the initial intermittent two-bottle choice procedure, animals later classified as “bingers” already displayed higher ethanol intake than those categorized as “Low drinkers”. This observation suggests that some degree of individual variability in alcohol preference may pre-exist before operant training. Such variability has been previously reported in outbred rodent populations, where stable individual differences in voluntary alcohol consumption can be observed even under relatively simple drinking paradigms [21, 22]. However, the operant binge-drinking procedure introduces additional behavioral dimensions that are not captured by the two-bottle choice paradigm, including response cost, temporal compression of intake, cue-controlled responding, and motivational effort. These contingencies allow the identification of phenotypes that differ not only in overall intake but also in behavioral features such as speed of consumption, cue sensitivity, relapse-like responding, and resistance to outcome devaluation. In this context, the operant paradigm may not generate these phenotypes de novo but rather reveal and amplify pre-existing vulnerability traits by placing animals in conditions where these behavioral differences become more readily expressed.

Among the four groups, there is group 1 of ‘Fast Bingers’ characterized by rapid consumption of large amount of alcohol, group 2 of ‘Bingers’ who drink similar large amount of alcohol but a little bit slower, group 3 of ‘Extreme bingers’ characterized by heavy alcohol consumption, and group 4 of ‘low drinkers’ characterized by lower motivation, seeking, and relapse. Low drinkers only had to perform 11 presses and thus to consume 3 rewards to achieve the 50% of their total amount whereas the extreme bingers had to perform 40 active lever presses to achieve their 50% of total amount. That certainly explain the speed of consumption for the ‘low drinkers’ group.

In contrast to the study of Wheeler et al. [23], which concluded that shortening session duration does not promote binge drinking, our operant self-administration paradigm relies on a fundamentally different behavioral framework. In their “sipper” model, a single response provides prolonged access to ethanol, resulting in low motor demand and limited engagement of repetitive or habit-like processes. By contrast, our procedure requires repeated responding (FR-3) to obtain small, discrete ethanol deliveries, thereby imposing a higher response cost and promoting a more structured, repetitive pattern of intake. Under these conditions, reducing the session duration produces a clear binge-like profile, characterized by rapid intake and elevated blood ethanol levels. More broadly, temporal compression has been shown to enhance front-loading behavior and can shift male intake toward patterns more typically observed in females under operant schedules [24]. Therefore, the conclusion that shortening session duration does not influence drinking behavior cannot be generalized across paradigms. Key procedural differences—particularly in ethanol delivery and response requirements—critically determine the behavioral outcomes and must be considered when interpreting these findings.

Binge drinking and AUD may be associated with habit learning development, and it is conceivable that habit learning among binge drinkers could play a role in the transition from binge drinking behavior to AUD. Previous studies have suggested that binge drinking is a repetitive behavior which can lead to the formation of habits and have also shown that student alcohol consumption is ‘automatically activated by relevant contextual cues’ [25]. Habitual binge drinking can be assessed with the Self-Report Habit Index that is composed of some items such as ‘I do frequently’, ‘I do automatically’, ‘I do without having to consciously remember’ and ‘I do without thinking’ [25]. High automaticity score has been associated with an increased risk of harmful alcohol use [26]. In animals, when operant behavior is unaffected by a loss of subjective value of alcohol (devaluation), the behavior is considered habitual. Interestingly, our results indicate that group 3 of ‘Extreme bingers’, predominantly females, tends to consume the most alcohol in a devaluation session and exhibit behavior of perseverance of alcohol consumption despite satiety. This initial analysis also revealed sex-related proportion differences among the groups, with an equal distribution of males and females in group 1, more males in groups 2 and 4, and more females in group 3 of ‘Extreme bingers’. This prompted us to conduct a more in-depth analysis of the results across the entire cohort, focusing on sex differences.

Sex-specific analyses show that females consume more alcohol (35% increase) with a slightly higher consumption speed (20% decrease of the latency to achieve 50% of their total intake). While there is no difference in motivation to consume, females exhibit higher alcohol-seeking behavior (36% increase) associated with higher relapse (+ 40%) and withdrawal scores (46% increase). It is also observed that the proportion of females significantly increases their alcohol-seeking behavior when the drug is omitted and that the number of active lever presses tends to be decreased when the cue associated with the drug is omitted. This aligns with previous rodent studies reporting that females consume more alcohol relative to their body weight and display higher levels of cue-mediated alcohol-seeking behaviors than male [24, 27–30]. An additional point that deserves consideration concerns the interpretation of the cue-omission and seeking tests. In our study, females showed greater persistence in responding when alcohol delivery was omitted and increased their operant responding during the seeking session, in which no alcohol was available. At first glance, the persistence observed during cue omission might appear inconsistent with a simple increase in sensitivity to alcohol itself. However, repeated pairings between alcohol delivery and the conditioned stimulus may have led the cue to acquire incentive motivational properties through associative learning processes. In this context, responding may be maintained not only by the expectation of alcohol, but also by the motivational value attributed to the cue predicting reward delivery.

From this perspective, the behavioral pattern observed in females suggests a stronger reliance on cue–reward associations and a greater attribution of incentive salience to alcohol-associated cues compared with males. This interpretation is supported by the higher proportion of females increasing their lever pressing across cue-sensitive tests. Consistent with this idea, previous studies using food rewards have shown that females may display enhanced or more rapid acquisition of stimulus-directed Pavlovian approach behaviors (i.e., sign-tracking) [31, 32], although other reports indicate similar levels between sexes [33].

Notably, the persistence of responding in females despite the absence or devaluation of the reward also raises the possibility that, in some individuals, behavior may have shifted toward a more habitual mode of control. Indeed, continued engagement with reward-predictive cues under outcome devaluation conditions is often interpreted as reflecting reduced goal-directed control and increased habit-based responding. Together, these findings suggest that females may be particularly prone to developing cue-driven and potentially inflexible patterns of alcohol-seeking behavior in this paradigm.

The lack of sex-related difference observed here is also in line with previous study that measured breakpoints in Long Evans rats, but in this study, lower breakpoint values were achieved (around 10) compared to the ones we obtained (around 20) [29] probably due to the duration of the session 30 min vs. 15 min, but also to the difference in the schedule of increment used in both studies and the type of alcohol solution (20% v/v for our study and 15% ethanol + 2% sucrose in Randall’s one). Our results also demonstrate that females, as expected, consume more alcohol when alcohol is available *ad libitum* in their home cage for devaluation in the satiety test. We did not investigate the effect of the estrous cycle on alcohol-related behaviors in our rats because it has been repeatedly demonstrated that estrous cycle does not substantially impact alcohol intake in naturally cycling rats [34–36].

While the rewarding effects of alcohol contribute substantially to its addictive potential, there is a growing acknowledgment that the aversive properties of alcohol also wield a crucial influence on the inclination to consume it. Following recurrent episodes of binge drinking, individuals may develop a negative affective state upon voluntarily or involuntarily withdrawal from alcohol. This encompasses dysregulated stress hormone levels, dysphoria, anxiety, depression, and irritability, an array of symptoms believed to stem, at least in part, from adaptations in stress-related neural pathways [37]. Across sexes, the withdrawal composite score clearly discriminated ethanol-exposed animals from saccharose controls, supporting its utility as a global index of abstinence-related signs. However, the pattern of sex differences depended on the ethanol exposure paradigm. In the operant cohort, females exhibited higher withdrawal scores than males, an effect mainly driven by tail stiffness and escape/jumping attempts. These behavioral components may reflect increased behavioral reactivity or aversive state during early abstinence rather than a uniformly more severe neuromotor withdrawal syndrome. In addition, it is interesting to note that males and females do not react similarly to individual housing as used in this study. Females tends to show increase in corticosterone levels in this condition as compared to males or social housing [30]. These differences could also contribute to the increase in alcohol consumption observed in our study. We showed that females displayed more severe withdrawal score and this result suggests that increased sensitivity to the aversive properties of alcohol withdrawal may contribute to higher levels of binge drinking in order to alleviate withdrawal symptoms. These results are in line with clinical observations suggesting that women are more likely to drink alcohol to alleviate unpleasant emotions and relapse in response to negative affect [38, 39]. To pursue on this line, our data reveal that females exhibit higher consumption than males during relapse after abstinence. Although we observed a higher level of ethanol relapse in females compared to males after 10 days of abstinence, another study conducted on Long Evans rats demonstrated the opposite trend, with no relapse behavior observed in females and a clear relapse in males after a 3-week period of abstinence [29]. However, in this later study, rats had access to alcohol during 30 min sessions and with sucrose added to the ethanol solution. We already demonstrated that, even with a similar level of alcohol self-administration, the speed of consumption is crucial to alter alcohol-associated behaviors [9]. After prolonged abstinence, alcohol-associated cues can acquire strong incentive salience, thereby intensifying cravings and precipitating relapse [12]. In humans, men generally report higher levels of alcohol craving than women [40], and similarly, male rodents are often described as more susceptible to relapse than females [41]. However, our findings point in the opposite direction since in our paradigm, females displayed more pronounced seeking behavior when alcohol was omitted, as well as higher drinking levels during relapse following abstinence.

A cue-omission session was used to evaluate the contribution of alcohol-associated cues to ongoing operant responding. In this paradigm, ethanol delivery remained available while the conditioned cues normally paired with reward were omitted. Therefore, this test does not correspond to a classical cue-induced reinstatement paradigm, which typically involves extinction followed by cue-triggered relapse. Instead, it probes the extent to which ongoing alcohol-reinforced responding is controlled by previously learned cue–reward associations. However, because ethanol reinforcement remained available and the instrumental contingency was unchanged, the cue-omission session primarily reflects the relative contribution of conditioned cues versus instrumental contingencies in maintaining alcohol-seeking behavior. In parallel, the drug-omission test used here differs from classical post-extinction drug-seeking procedure. It consists of a single session performer in animals with an established history of alcohol self-administration, in the same context previously associated with alcohol availability, but in which alcohol is unexpectedly withheld. As such, it may reflect context-induced alcohol seeking or craving-like responding rather than reinstatement after extinction. Importantly, alternative interpretations should also be considered. The absence of expected cues or of the reward may generate a form of prediction error or mild frustration, which could transiently modify responding.

In addition, our study revealed that females exhibit a reduced sensitivity to outcome devaluation, which can be interpreted as a form of behavioral inflexibility. In contrast, males almost completely ceased alcohol consumption following devaluation. Previous work has shown that female alcohol-preferring rats display a tendency toward habitual behavior, reflected for example by an increased latency to initiate reward responding after devaluation (cherry-flavored unsweetened solution), a recognized indicator of habit formation [42]. Together, these sex-related differences are particularly intriguing, as they suggest that females may be more prone to developing automatic, habit-based behaviors that could contribute to a greater susceptibility to AUD. However, this interpretation will need confirmation from clinical studies. Supporting this hypothesis, a recent study reported that female Wistar rats displayed higher levels of compulsive alcohol self-administration, both foot-shock-resistant and quinine-resistant behaviors that are commonly interpreted as reflecting a transition from goal-directed to habitual responding [43]. Similarly, another systematically devaluating alcohol by delaying its delivery found that females persistently engaged in habitual responding despite delay-induced reinforcer devaluation [34]. Overall, our results, in combination with findings from these previous studies, suggest that females may be more vulnerable to the development of habit learning and compulsive alcohol use, which aligns with clinical observations indicating that women may be particularly at risk for the rapid emergence of AUD.

### Perspective and significance

Preclinical models are essential for dissecting the behavioral and neurobiological mechanisms that underlie binge drinking. Recognizing sex as a critical biological variable is fundamental to identifying vulnerability factors contributing to the initiation and escalation of binge drinking behaviors. Our dataset provides a comprehensive characterization of how sex differences shape alcohol drinking patterns, offering valuable insights that can guide the development of improved prevention and treatment strategies. Moreover, as recently demonstrated by our group [44], pharmacological treatments for AUD may differ in efficacy between males and females, highlighting the necessity of systematically incorporating both sexes in preclinical and translational research on AUD.

## Conclusions

In conclusion, our study used unsupervised clustering analyses to identify distinct groups of binge drinking profiles, mirroring the heterogeneity observed in clinical populations. We identified four primary groups on multiple drinking dimensions. Our findings highlight the importance of resistance to reward devaluation as key feature of binge drinking behavior and a potential marker of vulnerability to AUD. We also observed clear sex-related differences across these groups, females exhibited stronger alcohol-seeking responses, higher withdrawal scores, and increased consumption during relapse compared with males. Furthermore, our data suggest that females may be more prone to developing cue-driven automatic behaviors, consistent with habit learning, which could contribute to their heightened susceptibility to AUD. Altogether, these findings provide important insights into the complex interplay between sex, drinking trajectories, and the development of compulsive alcohol use, and they underscore the need for continued investigation in clinical populations.

## Electronic Supplementary Material

Below is the link to the electronic supplementary material.

Supplementary Material 1

## Data Availability

The datasets used and/or analyzed during the current study can be made available from the corresponding author on reasonable request.
