# Supplementary material for: Female rats are more vulnerable to binge drinking behavior in an operant self-administration paradigm: implication for transition to alcohol use disorders
Source: Biol Sex Differ. 2026 Apr 29;17:91. doi: 10.1186/s13293-026-00912-x (PMC13130434; doi:10.1186/s13293-026-00912-x)

**Additional materials**

***Clustering analysis***

**Legend Additional Figure 1**: Unbiased clustering analysis. **A.** Values enabling adjustment of the best model obtained from the K-means clustering method. **B:** Elbow method for determining the number of clusters. **C:** Screen plot of the four clusters based on consumption (g/kg) and time to achieve 50% of total rewards.


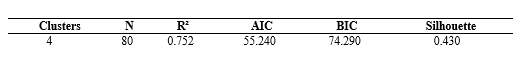

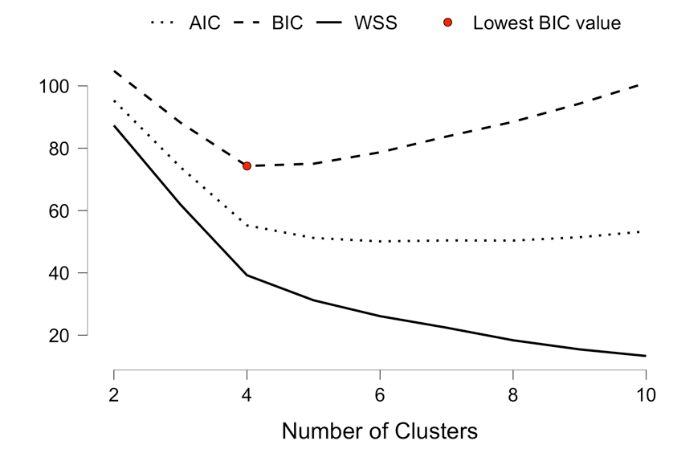

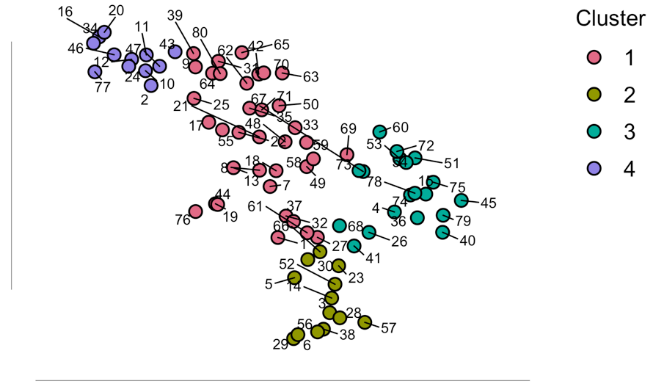


**Legend Additional Figure 2:** Unbiased clustering analysis done by sex (**A-D** for the Males and **E-G** for the Females). **A and E** represent the Values enabling adjustment of the best model obtained from the K-means clustering method. **B and F**: Elbow method for determining the number of clusters. **C and G** represents the proposition of individuals in each of the subgroups identified by the k-mean method. **D and H**: Screen plots of the four clusters based on consumption (g/kg) and time to achieve 50% of total rewards for each sex.


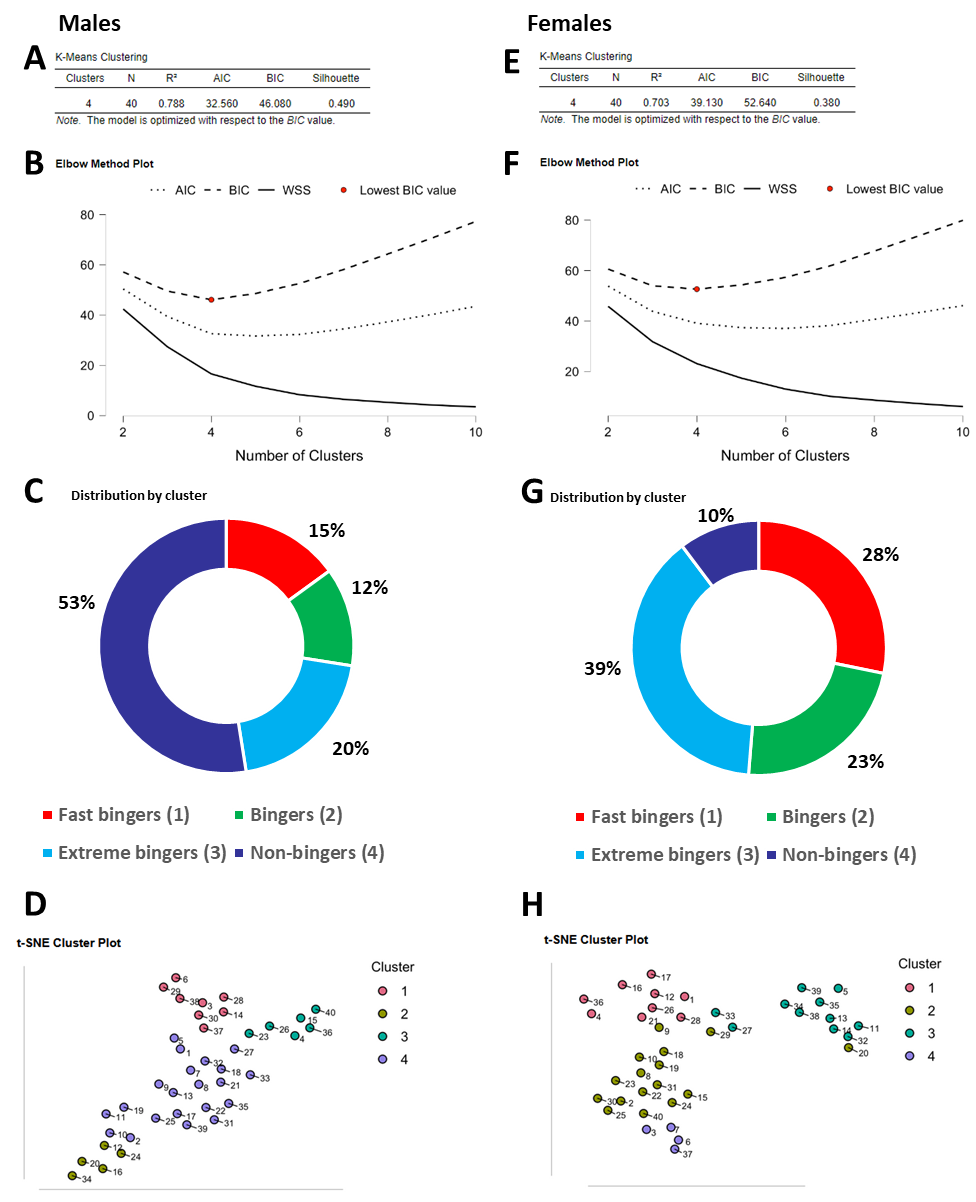


**Legend Additional Figure 3:** **Clustering analysis within the male group.** Based on the quantity of alcohol consumed (A) and the speed of consuming it (B), the clustering analysis provided 4 distinct groups of consumers: FB = Fast Bingers, B = Bingers, EB = Extreme bingers and LD = Low Drinkers. The operant responding (C) during a typical session of OBD was analyzed depending of these clusters, as well as the motivation (D) through a progressive ratio session, the seeking (E) for alcohol (a session in which alcohol is absent but the cues associated are present), the perseverance of operant responding during a session in which alcohol is delivered but the cue-associated are absent (F, cue omission) and finally the relapse (G) after 14 days of abstinence. The bold black dotted line within each violon box indicates the median and the fine dot-colored lines represent the quartiles. * p < 0.05; ** p < 0.01; *** p < 0.001

**Legend Additional Table 1:** Statistical analysis of each behavior depending on the clustering analysis within the male rats. The normality and equality of variances were tested. If validated, an ANOVA (F value in the table) followed by a Tukey test for multiple comparisons were performed on the data set. If not validated, a Kruskal-Wallis test followed by a Dunn test for multiple comparisons were performed. The results of the multiple comparisons are indicated in each panel of Additional Fig. 3. ns : not significant

| **AUD-associated behaviors** | **ANOVA or Kruskal Wallis** |
| --- | --- |
| **SA – Active lever presses** | **F_(3,36)_ = 15.03 p < 0.001** |
| **SA – EtOH consumed (g/kg/15 min)** | **H = 23.97 p < 0.001** |
| **Speed of consumption (time to achieve 50% of total consumption - min)** | **H = 28.41 p < 0.001** |
| **Motivation**  **(active lever presses)** | **H = 12.21 p < 0.01** |
| **Seeking**  **(active lever presses)** | **H = 7.8 p = 0.05** |
| **Cue Omission**  **(active lever presses)** | **F_(3,36)_ = 10.46 p < 0,001** |
| **Relapse**  **(g/kg/15 min)** | **F_(3,35)_ = 8.78 p < 0.001** |

**Legend Additional Figure 4: Clustering analysis within the female group.** Based on the quantity of alcohol consumed (A) and the speed of consuming it (B), the clustering analysis provided 4 distinct groups of consumers: FB = Fast Bingers, B = Bingers, EB = Extreme bingers and LD = Low Drinkers. The operant responding (C) during a typical session of OBD was analyzed depending of these clusters, as well as the motivation (D) through a progressive ratio session, the seeking (E) for alcohol (a session in which alcohol is absent but the cues associated are present), the perseverance of operant responding during a session in which alcohol is delivered but the cue-associated are absent (F, cue omission) and finally the relapse (G) after 14 days of abstinence. The bold black dotted line within each violon box indicates the median and the fine dot-colored lines represent the quartiles. * p < 0.05; ** p < 0.01; *** p < 0.001

**Legend Additional Table 2:** Statistical analysis of each behavior depending on the clustering analysis within the female rats. The normality and equality of variances were tested. If validated, an ANOVA (F value in the table) followed by a Tukey test for multiple comparisons were performed on the data set. If not validated, a Kruskal-Wallis test followed by a Dunn test for multiple comparisons were performed. The results of the multiple comparisons are indicated in each panel of Additional Fig. 4. ns : not significant

| **AUD-associated behaviors** | **ANOVA or Kruskal Wallis** |
| --- | --- |
| **SA – Active lever presses** | **H = 27.02 p < 0.001** |
| **SA – EtOH consumed (g/kg/15 min)** | **F_(3,36)_ = 28.77 p < 0.001** |
| **Speed of consumption (time to achieve 50% of total consumption - min)** | **H = 29.50 p < 0.001** |
| **Motivation**  **(active lever presses)** | **H = 7.33 ns** |
| **Seeking**  **(active lever presses)** | **H = 6.51 ns** |
| **Cue Omission**  **(active lever presses)** | **F_(3,36)_ = 4.03 p < 0,05** |
| **Relapse**  **(g/kg/15 min)** | **F_(3,36)_ = 3.21 p < 0.05** |

**Legend Additional Table 3: Item-wise sex differences in withdrawal signs in the operant ethanol cohort.** For each withdrawal criterion (vocalizations/aggression, freezing at cage opening, tail stiffness, escape/jumping attempts, stereotypies/tremors), values are reported separately for females and males as mean ± SD (scores range 0–2). Sex differences were tested using two-sided Mann–Whitney U tests (non-parametric, appropriate for ordinal item scores), with unadjusted p-values and multiplicity-adjusted p-values (Holm correction) reported to control family-wise error across the 5 items. This table identifies which specific withdrawal components contribute to the sex effect observed in the total withdrawal score. In bold are represented the significant values of p < 0.05.

| Item | Female mean±SD | Male mean±SD | p (MW) | p_adj (Holm) |
| --- | --- | --- | --- | --- |
| Vocalizations | 0.225 ± 0.577 | 0.225 ± 0.480 | 0.6569 | 0.6569 |
| Freezing at cage opening | 0.000 ± 0.000 | 0.100 ± 0.304 | **0.04273** | 0.1282 |
| Tail stiffness | 0.775 ± 0.423 | 0.475 ± 0.554 | **0.005857** | **0.02343** |
| Escape/jumping attempts | 0.350 ± 0.483 | 0.050 ± 0.221 | **0.0008806** | **0.004403** |
| Stereotypies/tremors | 0.825 ± 0.636 | 0.575 ± 0.594 | 0.07703 | 0.1541 |

**Legend Additional Figure 5: Total withdrawal score in operant ethanol–exposed rats and sucrose controls (sex comparison).**

Total withdrawal score (sum of 5 items; range 0–10) was assessed after 5 days of abstinence. Boxplots show the median (central line), interquartile range (box), and whiskers extending to 1.5×IQR; open circles indicate outliers. Colors indicate sex (female: pink; male: blue), with lighter shades for sucrose controls and darker shades for ethanol (operant self-administration) animals. Within each condition (sucrose or ethanol), females are displayed on the left and males on the right. P-values above brackets correspond to two-sided Mann–Whitney tests comparing females vs males within each condition.


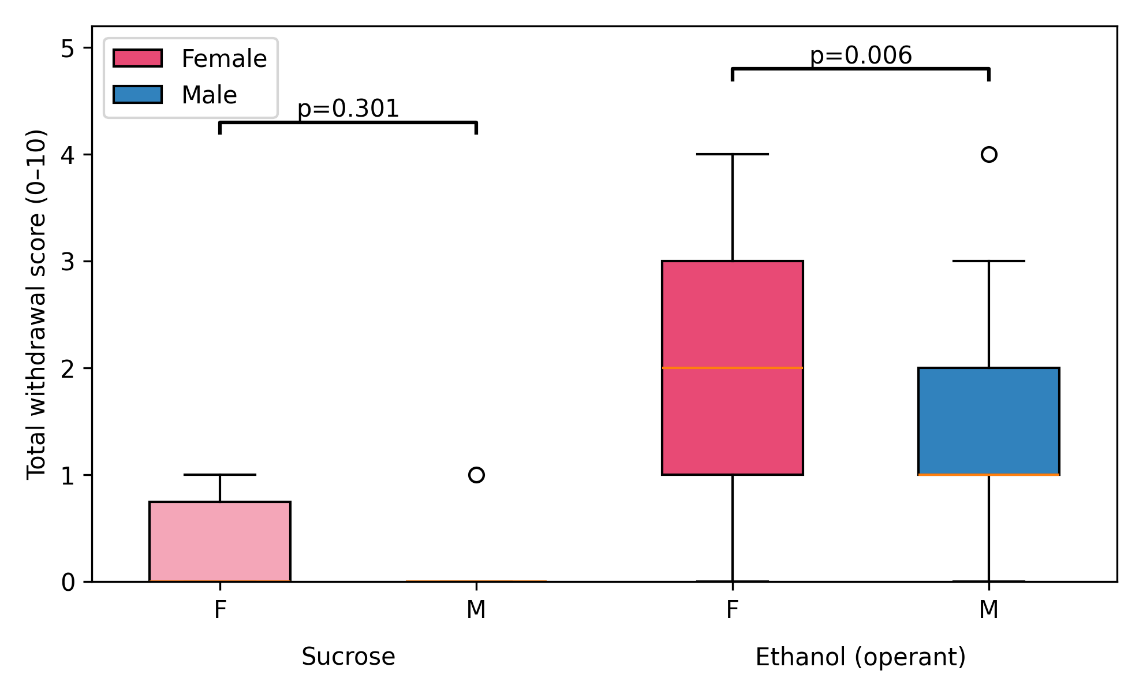


**Legend Additional Figure 6:** **Item-wise distribution of withdrawal scores in the operant ethanol cohort (female vs male).**

Stacked bar plots represent the proportion of animals scoring 0, 1, or 2 for each withdrawal criterion in ethanol-exposed rats (operant self-administration). For each criterion, females are displayed on the left (F) and males on the right (M). Shading encodes symptom intensity: score 0 (absence; light grey), score 1 (moderate; medium grey), and score 2 (high; dark grey). Item labels are centered beneath each female/male pair to facilitate direct comparison. P-values displayed above each criterion correspond to two-sided Mann–Whitney tests comparing female vs male item scores (ordinal 0–2).


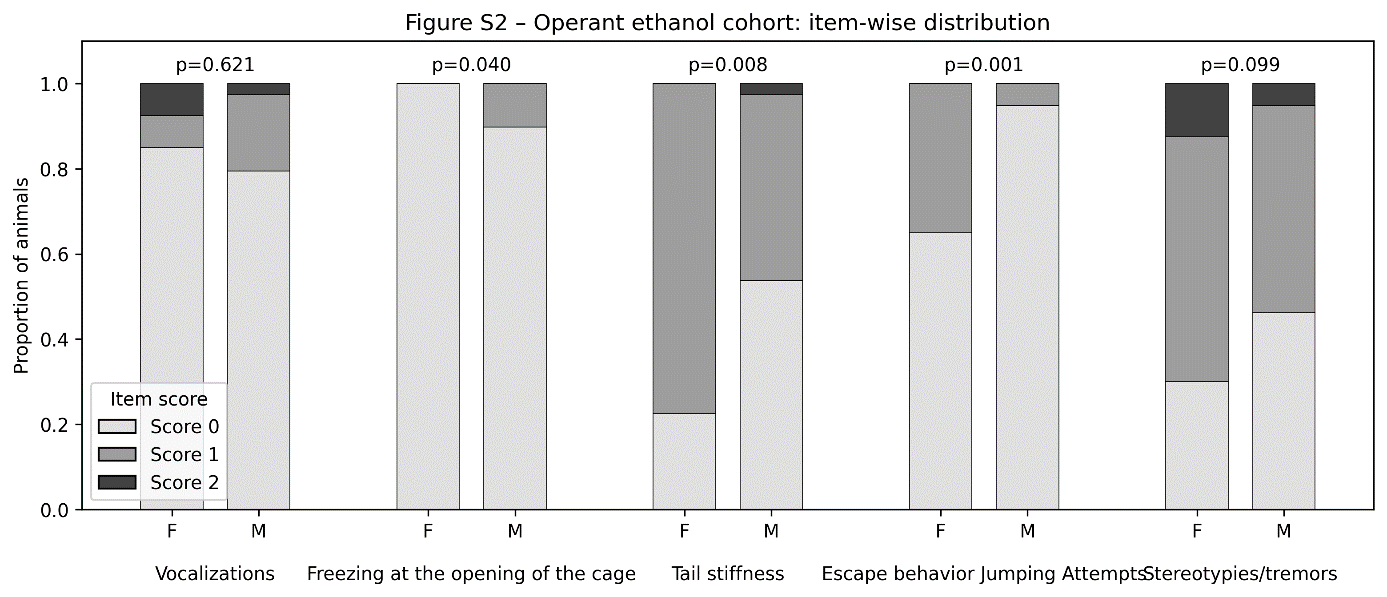


**Legend Additional Figure 7:** **Item-wise distribution of withdrawal scores in saccharose controls (female vs male).**

Stacked bar plots represent the proportion of animals scoring 0, 1, or 2 for each withdrawal criterion in saccharose-only control rats. For each criterion, females are displayed on the left (F) and males on the right (M). Shading encodes score intensity: 0 (light grey), 1 (medium grey), 2 (dark grey). Item labels are centered beneath each female/male pair. P-values displayed above each criterion correspond to two-sided Mann–Whitney tests comparing female vs male item scores (ordinal 0–2). As expected for non-ethanol-exposed controls, scores are near-zero across items, supporting the specificity of the withdrawal scoring system.


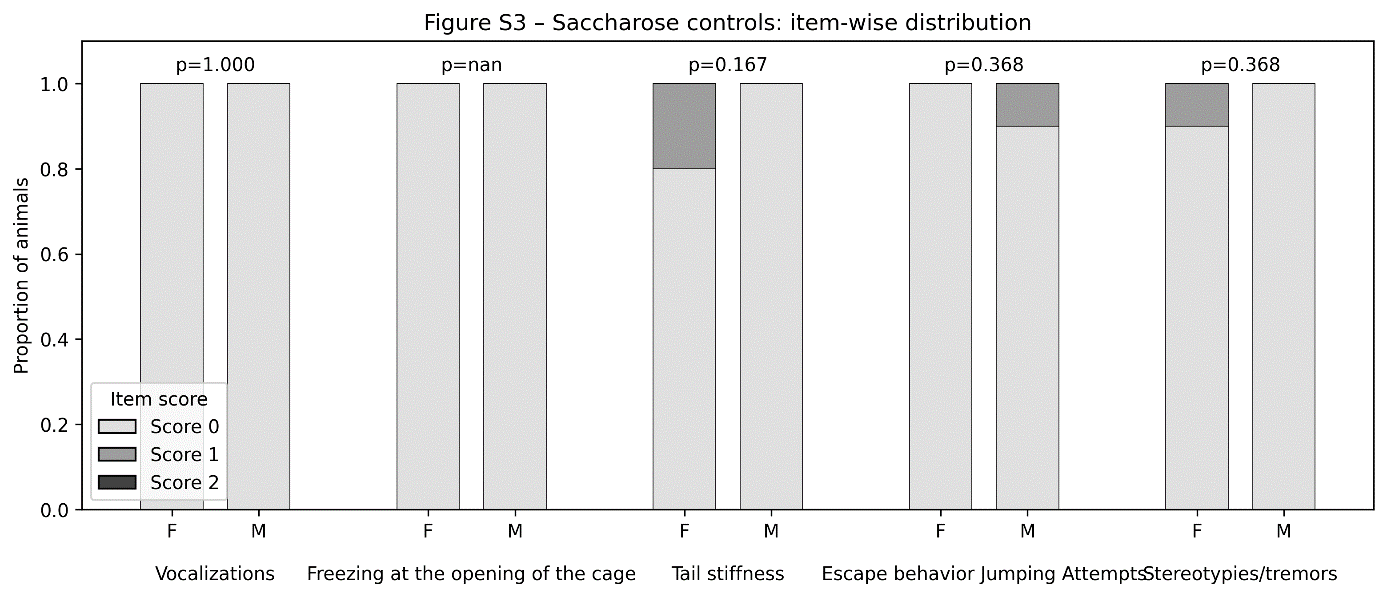


**Legend Additional Figure 8: Correlations between the withdrawal scores and either the total number of active lever presses or the speed of consumption. A - B)** Correlations within the females; **C-D)** Correlations within the males; **E-F)** Correlations both sexes mixed together. Results are analyzed using a Pearson test.


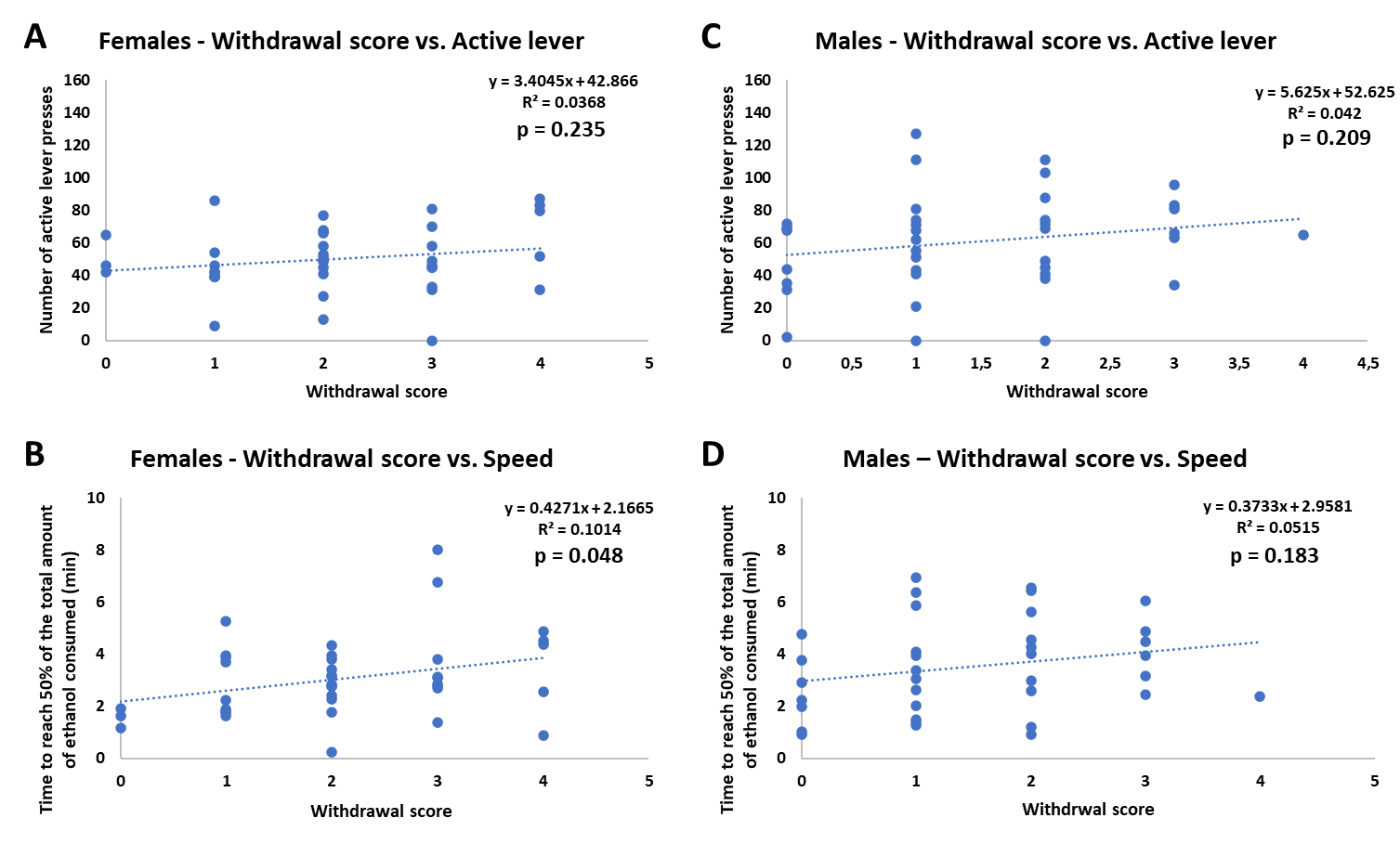

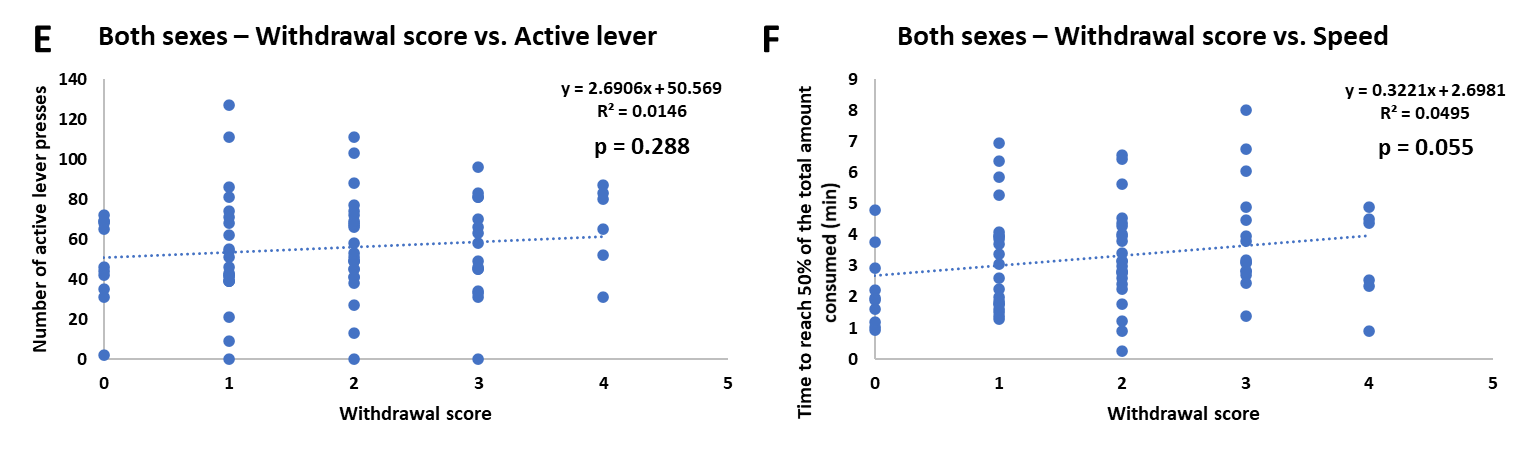

Supplement: Supplementary file 1 — Supplementary Material 1 [file 13293_2026_912_MOESM1_ESM.docx]
